# Effects of High-Altitude Environments on Gut Microbiota and Their Mechanisms in Immune Regulation and High-Altitude Adaptation

**DOI:** 10.3390/ijms27115096

**Published:** 2026-06-04

**Authors:** Zhipeng Lu, Guojing Chen, Mingyang Chang, Ningning Wang, Tiantian Xia, Yunan Zhang, Gaoyuan Xu, Qianqian Zhao, Pan Shen, Wei Zhou, Zhexin Ni, Yue Gao

**Affiliations:** Academy of Military Medical Sciences, Beijing 100850, China; luzhipeng316@163.com (Z.L.); muyufuchen_1@163.com (G.C.); chmingyang@163.com (M.C.); wnnbeijing@163.com (N.W.); xiatianttx@163.com (T.X.); 20210931173@bucm.edu.cn (Y.Z.); 15563478012@163.com (G.X.); 15358865713@163.com (Q.Z.); spluto@foxmail.com (P.S.); zhouweisyl802@163.com (W.Z.)

**Keywords:** high-altitude environment, gut microbiota, disease mechanisms, immune regulation, high-altitude adaptation

## Abstract

High-altitude environments, characterized by hypoxia, low temperature, and intense ultraviolet radiation, profoundly disrupt host intestinal homeostasis and reshape the gut microbiota, thereby influencing immune regulation and acclimatization. This review systematically summarizes the dynamic compositional and functional changes in the gut microbiota in high-altitude natives, immigrant populations, short-term visitors, and relevant animal models. Current evidence indicates that long-term high-altitude adaptation is associated with directional microbial remodeling, including the enrichment of anaerobic and short-chain fatty acid (SCFA)-associated taxa, which may support energy metabolism and immune homeostasis. In contrast, acute high-altitude exposure more readily induces dysbiosis, impairs intestinal barrier integrity, and promotes the translocation of endotoxins and bioactive metabolites. Mechanistically, the gut microbiota and its metabolites participate in high-altitude adaptation and high-altitude-related disease pathogenesis by modulating barrier function, inflammatory responses, oxidative stress, and immune signaling, and by mediating interorgan communication—characterized by metabolite-driven systemic inflammation or tolerance—through the gut–lung, gut–heart, gut–brain, gut–kidney, and gut–testis axes. SCFAs, bile acids, amino acid-derived metabolites, and succinic acid may control immune homeostasis and inflammatory responses through pathways including TLR4/NF-κB and NLRP3. Although the causal relationships, core microbial effectors, and population-specific heterogeneity remain incompletely defined, microbiota-targeted interventions, including probiotics, prebiotics, and fecal microbiota transplantation, have shown promise for promoting acclimatization and preventing high-altitude-related disorders. Overall, this review provides an integrated framework linking environmental stress, gut microbial ecology, and host immune–metabolic adaptation at high altitude, and highlights future directions for mechanistic and translational research in high-altitude medicine.

## 1. Introduction

High-altitude regions possess unique geographic and climatic characteristics, such as low barometric pressure, hypoxia, severe cold, strong ultraviolet radiation, and dryness, which pose severe challenges to human physiological homeostasis. It is estimated that approximately 2% of the global population lives in high-altitude areas above 1500 m, and these special environments induce a series of adaptive physiological changes in high-altitude residents concerning body morphology, respiratory metabolism, cardiovascular hemodynamics, and immune regulation [1]. For instance, acute and chronic hypoxia can trigger inflammatory and endothelial responses relevant to acute mountain sickness, high-altitude pulmonary edema, and high-altitude cerebral edema [2]. Furthermore, the cardiovascular system in high-altitude areas also faces substantial stress, manifested as enhanced sympathetic nerve excitability, increased heart rate, and elevated blood pressure; during long-term adaptation, cardiac structural remodeling and hemodynamic parameter adjustments occur [3,4]. Although these physiological changes help to cope with the hypoxic environment, they also increase the risk of cardiovascular diseases and other high-altitude illnesses. Notably, the gastrointestinal tract is an organ extremely sensitive to hypoxia and ischemia. High-altitude hypoxic environments can easily cause intestinal microcirculatory disorders, leading to impaired intestinal mucosal barriers and increased permeability, providing a critical entry point for the study of intestinal microecology [5].

As an important regulatory factor for host physiological health, the gut microbiota has been extensively studied in recent years for its potential role in environmental adaptation. The gut microbiome not only participates in host nutritional metabolism and immune regulation but also regulates intestinal barrier function and systemic inflammatory responses by producing metabolites such as short-chain fatty acids (SCFAs; including acetate, propionate, and butyrate), which are essential fermentation products that regulate host energy homeostasis and immune responses [6,7]. Under high-altitude exposure, the diversity and function of the host gut microbiota undergo significant changes. For instance, studies have found that the diversity and structure of the gut microbiota in high-altitude residents differ from those in low-altitude populations, and abundance changes in certain core genera, such as *Akkermansia muciniphila*, suggest that high-altitude environments may shift the intestinal microecology in a direction unfavorable to host metabolic homeostasis [8,9]. Animal model studies have also confirmed that hypoxia and cold environments can drive the remodeling of the microbiota’s functional metabolic pathways to adapt to special energy demands [10]. Additionally, by regulating host bone metabolism, inflammatory responses, and energy metabolism, the gut microbiota may participate in high-altitude adaptation and the pathogenesis of high-altitude diseases [11,12]. Synthesizing recent cross-species microecological research, the directional remodeling of the gut microbiota in response to high-altitude environments is not merely a concomitant biomarker of stress, but a core pathophysiological driver mediating altitude-related pathologies [13]. In this context, gut–organ axes refer to bidirectional communication between gut microbiota and distant organs, such as the lung, heart, and brain, mainly through microbial metabolites, immune mediators, and neuroendocrine signals.

Studying the interaction between high-altitude environments and gut microbiota holds significant scientific and clinical value. First, understanding microbiota changes under high-altitude exposure may offer a useful framework for interpreting host responses to hypoxic and cold stress through microbial community adjustments, providing a new perspective on the molecular and metabolic mechanisms of high-altitude adaptation [14]. Second, as an important mediator regulating immunity and metabolism, the imbalance of gut microbiota is closely related to high-altitude-related diseases such as high-altitude pulmonary edema, high-altitude cerebral edema, and cardiovascular diseases; analyzing its action mechanisms facilitates the development of microbial intervention strategies to promote the health of high-altitude residents [15].

However, several critical limitations in the existing literature remain to be addressed. First, while genomic mapping has generated a wealth of descriptive data on microbial composition, a synthesized view linking these taxa to functional metabolic pathways remains fragmented. Moreover, previous reviews have not fully integrated microbial compositional changes, microbial metabolites, immune signaling pathways, and gut–organ axes into a coherent framework. In view of this, this review systematically reviews recent research progress on the dynamic evolution of gut microbiota under high-altitude environmental exposure, focusing on analyzing the potential mechanisms by which the gut microbiota regulates host immune responses, participates in the establishment of high-altitude adaptation, and mediates multi-organ axis interactions through its metabolites. This review aims to provide a new theoretical perspective for cross-disciplinary research in high-altitude medicine and microecology, and to offer a scientific basis for the future development of strategies for the prevention and treatment of high-altitude diseases based on targeted gut microbiota interventions (such as microecological preparations) [16].

A structured literature search was performed to identify studies related to high-altitude exposure, gut microbiota, immune regulation, and high-altitude adaptation. Relevant articles were searched in PubMed, Web of Science, Scopus, and Google Scholar using combinations of terms such as “high altitude,” “hypoxia,” “gut microbiota,” “microbiome,” “immune regulation,” “inflammation,” “intestinal barrier,” “short-chain fatty acids,” and “high-altitude disease.” Human studies, animal studies, experimental hypoxia models, mechanistic studies, and microbiota-targeted intervention studies were included when they reported microbiota-related changes under high-altitude or hypoxic conditions. Studies were excluded if they were not directly related to high-altitude or hypoxia, did not assess gut microbiota, or lacked relevance to host adaptation, immune regulation, metabolism, or high-altitude-related disease. As this review is narrative in nature, the selected studies were used to summarize current evidence, identify major mechanisms, and discuss existing inconsistencies and future directions.

**Figure 1:**
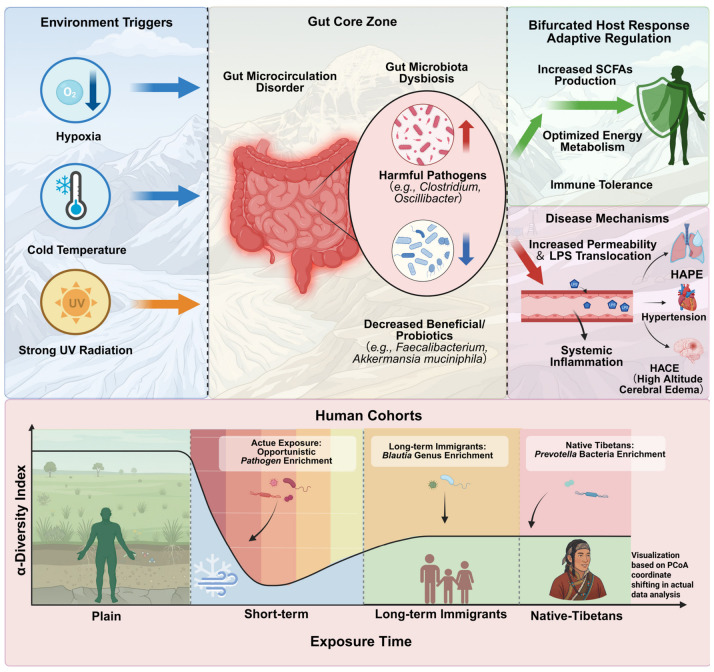
Schematic overview of high-altitude-driven gut microbiota remodeling and its stage-dependent dynamics across human exposure cohorts. Core environmental stressors at high altitude, including hypoxia, cold temperature, and intense ultraviolet radiation, may induce gut microcirculatory disturbance and microbiota dysbiosis, characterized by expansion of opportunistic/pathogenic taxa (e.g., *Clostridium* and *Oscillibacter*) and depletion of beneficial commensals/probiotics (e.g., *Faecalibacterium* and *Akkermansia muciniphila*). These alterations lead to a bifurcated host response: on the adaptive side, microbial metabolic reprogramming may increase short-chain fatty acid (SCFA) production, optimize energy metabolism, and promote immune tolerance; on the pathogenic side, increased intestinal permeability and lipopolysaccharide (LPS) translocation may trigger systemic inflammation and contribute to high-altitude pulmonary edema (HAPE), hypertension, and high-altitude cerebral edema (HACE). The (**lower panel**) schematically depicts the temporal trajectory of microbial restructuring across plain residents, short-term entrants, long-term immigrants, and native Tibetans, with an early decline in α-diversity and opportunistic-pathogen enrichment, followed by adaptive enrichment of *Blautia* in long-term immigrants and *Prevotella* in native highlanders. Created in BioRender. ZP, L. (2026) https://BioRender.com/dmb08vy (accessed on 21 April 2026).

To provide a conceptual framework for the following sections, the environmental factor-driven gut microbiota remodeling and its temporal dynamics across different human exposure cohorts are schematically presented in Figure 1.

## 2. Effects of High-Altitude Environments on the Gut Microbiota of Different Populations

### 2.1. High-Altitude Natives

The gut microbiota of high-altitude natives exhibits unique structural characteristics, primarily reflected in changes in microbial diversity and dominant taxa. Studies have shown that the gut microbiome of Qinghai–Tibet Plateau residents is influenced by extreme environmental factors such as high-altitude hypoxia, low temperatures, and strong ultraviolet radiation, leading to significant changes in gut microbiota composition. For example, the high-altitude environment promotes an increase in the abundance of strict anaerobes while reducing the number of aerobes and facultative anaerobes; this shift is conducive to increasing the proportion of probiotics and inhibiting the growth of pathogenic bacteria, reflecting the crucial role of gut microbiota in high-altitude adaptation [17]. The gut microbiota of native Tibetans exhibits a significant altitudinal gradient structure; as altitude increases, microbial diversity increases, with *Prevotella* dominating, accompanied by the enrichment of butyrate-producing bacteria and facultative anaerobes, suggesting that the microbiome participates in high-altitude hypoxic adaptation by enhancing the production of SCFAs [18]. Furthermore, at the same altitude (3900 m), the fecal microbial diversity of native Tibetans was significantly higher than that of Han immigrants; butyrate-producing bacteria were enriched and positively correlated with platelet counts, and the functional potential of the gut microbial community in native Tibetans was closely related to carbohydrate metabolism, suggesting that the gut microbiota may participate in the high-altitude acclimatization process of the Tibetan population [19].

Factors influencing the gut microbiota structure of high-altitude residents mainly include dietary habits, genetic background, and environmental stress. Regarding diet, high-altitude residents primarily consume a high-fiber, high-protein roughage diet; for instance, the Tibetan diet includes a large intake of highland barley and yak meat, which are rich in fiber and protein, promoting an increased abundance of fiber-degrading microbes such as *Bacteroides* and *Prevotella*, thereby enhancing efficient energy utilization and metabolite production. Other studies have shown that the α-diversity of the gut microbiota in Tibetan coronary artery disease (CAD) patients at high altitudes is higher, with significant enrichment of SCFA-producing bacteria such as *Catenibacterium*, *Holdemanella*, and *Prevotella*, which positively correlates with the intake of fermented dairy products and dietary fiber, suggesting that traditional high-altitude diets may participate in disease modification by shaping unique microbial characteristics [20]. Beyond environmental factors, host genetic architecture significantly contributes to the stratification of the gut microbiota. For instance, distinct genetic discrepancies between Tibetan and Han populations—particularly those related to hypoxic adaptation loci—have been implicated in modulating host–microbiota co-adaptation, underscoring the active participation of host genomic backgrounds in directing the assembly of population-specific microbial ecologies [21].

Notably, this population-specific microbial ecology in high-altitude natives may deeply interact with their unique genetic background shaped by long-term evolutionary selection. Population genetic studies have confirmed that the adaptation of native Tibetans to hypoxic environments involves strong selective sweeps, particularly the acquisition of a specific EPAS1 haplotype derived from the introgression of archaic Denisovan-like DNA, alongside the adaptive evolution of hypoxia-responsive genes such as EGLN1. Such intrinsic host genomic adaptations may co-evolve with the enrichment of *Prevotella* and butyrate-producing bacteria—potentially by modulating epithelial metabolism or mucosal immunity—thereby establishing a distinct host–microbiota co-adaptation framework in native highlanders [22,23].

### 2.2. High-Altitude Immigrant Populations

When new immigrants first enter high-altitude environments, their gut microbiota structure undergoes significant adjustments due to hypoxia, cold, and changes in dietary structure. For instance, a longitudinal tracking study of 45 lowland Han males over 108 days found that within 24 days of entering a high altitude, microbial diversity decreased while functional redundancy increased; the genus *Blautia* within the phylum *Firmicutes* rapidly enriched and maintained a dominant position in the long-term high-altitude population. This bacterium mitigates hypoxia-induced intestinal inflammation and pulmonary hypertension by producing butyrate and synthesizing cobalamin; intragastric administration in mice can elevate SpO_2_ and improve the intestinal barrier [24]. These initial changes involve not only the replacement of bacterial species but also dynamic shifts in microbial functions, such as enhanced energy metabolism capacity and regulated metabolite synthesis, providing energy support and immune protection for the host.

As new immigrants prolong their stay in high-altitude environments, their gut microbiota gradually stabilizes and exhibits adaptive reorganization. A study comparing 393 males across an altitudinal gradient noted that high-altitude hypoxia could rapidly (within 4 days) and persistently (over 3 months) significantly reduce the gut microbial diversity of Han individuals, and as the time spent living at high altitudes extended, the general characteristics and clinical indicators of the intestinal microflora in the Han population increasingly resembled those of the Tibetan population. The gut microflora of Han individuals who returned to the plains for 3 months remained unrecovered, revealing that high-altitude environments exert a directional and enduring remodeling effect on the microbiota and host metabolism [25]. Furthermore, a recent longitudinal study further demonstrated that when healthy young and middle-aged populations migrated from low altitudes to 4500 m and were exposed for 6 months, the α-diversity of their gut microbiota significantly decreased, with an increase in *Actinobacteriota* and a decrease in *Bacteroidota*; genera such as *Blautia*, *Bifidobacterium*, *Fusicatenibacter*, and *Anaerostipes* were enriched, accompanied by extensive metabolic profiling remodeling dominated by lipid and organic acid derivatives, indicating that continuous high-altitude exposure can participate in long-term host adaptation through microbiota-metabolism synergistic reprogramming [26]. Meanwhile, in moderate-altitude (approx. 2900 m) populations, the gut microbiota can transition from a *Blautia*-dominated enterotype to a *Bacteroides*-dominated enterotype; this enterotype transition is correlated with decreased inflammation levels, remodeling of amino acid/fatty acid/bile acid metabolism, and improved fasting blood glucose, illustrating that altitude exposure not only affects microbial composition but may also regulate host metabolic phenotypes by driving dominant enterotype restructuring [27]. This adaptation process is reflected not only in compositional changes in the microbiota but also involves the co-evolution of the microbiota with host metabolic and immune functions; gut microbes promote long-term host adaptation to high-altitude environments by regulating host gene expression and metabolic pathways.

### 2.3. Short-Term Visitors

Through longitudinal tracking of 406 healthy Han males, it was found that acute high-altitude exposure (4500 m, 7 days) can significantly elevate opportunistic pathogens (*Ruminococcus*, *Oscillibacter*) and decrease SCFA-producing bacteria (*Faecalibacterium*, *Roseburia*, *Bifidobacterium*); changes in microbial β-diversity persist even after returning to low altitudes. Functionally, acute phase enrichment involves xenobiotic degradation pathways, shifting towards energy and vitamin metabolism after long-term exposure, suggesting that the gut microbiota participates in high-altitude hypoxic adaptation [28]. The gut microbiota is highly susceptible to interference from multiple stresses in high-altitude environments, such as hypoxia, cold, and ultraviolet radiation, manifesting as structural imbalance and functional abnormalities of the microbiota. This imbalance not only affects nutritional metabolism but may also induce intestinal diseases such as irritable bowel syndrome (IBS); studies have found that high-altitude environment-induced gut microbiota imbalance is closely associated with the occurrence of IBS, and the longer the high-altitude residence, the more the gut microbiota structure tends to stabilize, leading to significant relief of IBS symptoms [29]. Therefore, maintaining gut microbiota homeostasis is of great significance to the health of short-term visitors, and reasonable dietary interventions and probiotic supplementation may serve as important strategies to improve gut microbiota function and enhance immune defense [30].

Taken together, current human studies indicate that high-altitude exposure induces both rapid perturbation and longer-term remodeling of the gut microbiota, with distinct patterns observed in native high-altitude populations, immigrants, and short-term visitors. Representative human studies, including their major microbial, metabolic, and clinical findings, are summarized in Table 1. Although these human cohorts provide essential longitudinal data, clinical confounding factors and limited tissue accessibility restrict causal insights. To decipher the precise cellular and systemic pathways driven by high-altitude hypoxia, researchers have increasingly utilized standardized experimental animal models and native wildlife, as detailed below.

## 3. Effects of High-Altitude Environments on the Gut Microbiota of Different Animals

### 3.1. Experimental Animal Models Simulating High-Altitude Environments

In high-altitude environmental research, the selection of animal models is crucial for revealing the mechanisms of gut microbiota in high-altitude adaptation. As traditional experimental animals, mice possess clear genetic backgrounds and are easily operable and controllable, making them suitable for simulated high-altitude hypoxia and gut microbiota intervention studies. For instance, by housing C57BL/6 mice in a hypobaric hypoxic environment and combining them with probiotics such as the Tibetan-derived *Lactobacillus johnsonii* YH1136 strain, the protective role of the gut microbiota against high-altitude hypoxia-induced intestinal barrier damage can be effectively explored, revealing potential microbial-host interaction mechanisms and miRNA regulatory networks [31]. Furthermore, acute simulated high-altitude hypoxia animal experiments showed that after 24 h of exposure to a hypobaric hypoxic environment equivalent to 5500 m, the gut microbiota diversity of mice significantly increased, the *Firmicutes*/*Bacteroidetes* ratio decreased, and genera such as *Akkermansia*, *Parabacteroides*, and *Bacteroides* were enriched, while beneficial bacteria such as Bifidobacterium decreased, accompanied by enhanced potential pathogenic and stress-tolerance phenotypes, indicating that acute hypoxia can rapidly drive the intestinal microecology to remodel toward a stress-adaptive state [32]. Concurrently, a rat model of combined intermittent cold and hypobaric hypoxia exposure further demonstrated that pure hypoxia could induce an increase in *Enterobacteriales*, a decrease in *Lactobacillales*, accompanied by weight loss and elevated inflammation-related indicators, whereas the combined effect of cold and hypoxia could lower the *Bacteroides*/*Firmicutes* ratio, correct partial microbial imbalances, and buffer hypoxia-induced physiological abnormalities, indicating that the regulation of gut microbiota and host adaptation by complex high-altitude environmental factors features dual characteristics of synergy and balance [33].

### 3.2. High-Altitude Native and Wild Animal Models

As a typical large high-altitude ruminant, the adaptation studies of the yak gut microbiota help in understanding the energy metabolism and immune regulation mechanisms of large high-altitude mammals. The yak gut microbiota is dominated by *Firmicutes* and *Bacteroides*, possessing high diversity and complex microbial networks capable of promoting cellulose degradation and efficient energy utilization [34]. When the yak gut microbiota adapts to seasonal nutritional changes at high altitudes, the production of volatile fatty acids (VFAs), especially acetate and propionate, increases significantly; these metabolites provide a highly efficient energy source for the host, supporting its maintenance metabolism under low temperature and hypoxic conditions [35]. Concurrently, the structural adjustments of the gut microbiota promote an enhancement in cellulose degradation capacity, increasing the efficiency of energy release from roughage [36]. In addition, multi-omics approaches on native high-altitude ruminant models such as Tibetan goats have revealed the adaptation mechanisms of their liver–gut axis, indicating that the gut microbiome is closely linked to host metabolism and immune functions, promoting the animals’ survival under high-altitude hypoxia and high radiation environments [37].

Research indicates that wild animals living at high altitudes, such as the plateau pika, also exhibit significant changes in gut microbiota diversity. Compared with low-altitude individuals, high-altitude pikas have reduced gut microbiota diversity, weakened community complexity, and altered bacterial community ratios shifting from *Firmicutes* to *Bacteroides*, reflecting the adaptive adjustments of the microbial community to extreme environments [38,39]. Furthermore, studies on migratory birds to the Qinghai–Tibet Plateau, such as the Eurasian tree sparrow, show that the gut microbiota experiences dynamic changes related to season and altitude; beneficial bacteria such as Lactobacillus increase significantly in winter, an adjustment that helps promote thermogenesis and energy conservation, thereby enhancing the host’s cold tolerance [40]. Concurrently, cellulose and hemicellulose degradation-related genes and metabolic pathways in the intestines of high-altitude animals are significantly upregulated, promoting the decomposition of herbaceous polysaccharides and energy acquisition, proving that the functional enhancement of the gut microbiota supports host energy metabolism demands [41]. The high-altitude environment also promotes the convergent evolution of the gut microbiota [42]; wild macaques, humans, and dogs in high-altitude environments exhibit an increased number of core shared ASVs in their gut microbiota, and the microbial structures tend to converge, demonstrating that environmental pressure and diet collectively drive the adaptive reorganization of the gut microbiota [43]. In domestic animals, such as the Tibetan pig, the gut microbiota of high-altitude animals often shows more complex interaction networks and enrichment of energy-harvesting functions, which may be associated with metabolic and immune adaptation [44]. Moreover, extreme seasonal changes and anthropogenic factors in high-altitude environments also affect the composition and metabolic characteristics of the gut microbiota, as seen in the white-lipped deer, where the gut microbiota enhances SCFA production during the cold winter to promote immune regulation and cope with environmental stress [45]. Overall, the high-altitude environment, by altering microbial community structures and enriching key functional bacteria and metabolic pathways, promotes host energy metabolism optimization and immune homeostasis maintenance in harsh environments, reflecting the critical role of gut microbiota in high-altitude adaptation [46].

Furthermore, when interpreting microbiome variations in animal models, the confounding effect of diet must not be overlooked. Standardized laboratory chow differs drastically from the natural, high-fiber, or high-protein diets of wild high-altitude animals (such as yaks and pikas). Dietary shifts during altitude transition—even in short-term animal experiments—can fundamentally alter the substrate availability for microbial fermentation, directly impacting the expansion of specific taxa independent of hypoxic stress.

Compared with human observational studies, animal and cross-species investigations provide stronger experimental support for causal links between environmental stress, microbial remodeling, and host adaptation (Table 2).

While a consensus exists regarding the profound impact of high-altitude environments on the gut microbiota, it is crucial to acknowledge several contradictory findings in the current literature. The most prominent inconsistency lies in the alterations of microbial α-diversity. For instance, while some studies report an increase or higher baseline of α-diversity in high-altitude natives (such as Tibetans) and certain wild animals (like macaques), multiple longitudinal studies observe a significant decrease in diversity during acute or sub-acute high-altitude exposure in lowland immigrants. Similarly, findings regarding specific taxonomic shifts, such as the *Firmicutes*-to-*Bacteroidetes* ratio, remain conflicting across different animal models and human cohorts.

These discrepancies are unlikely to be simple experimental errors, but rather reflect the complex, multi-factorial nature of gut microecology. They likely stem from variations in exposure duration (acute hypoxia vs. evolutionary chronic adaptation), host genetic backgrounds, species-specific physiological traits, and strong confounding factors such as dietary differences (e.g., high-fiber traditional Tibetan diets vs. standardized laboratory feed). Furthermore, differences in experimental methodologies, including targeted amplicon sequencing versus shotgun metagenomics, may also contribute to the inconsistent taxonomic resolutions. Therefore, interpreting high-altitude microbiome data requires cautious consideration of these contextual and methodological variables.

## 4. The Role of Gut Microbiota in the Mechanisms of High-Altitude Environment-Related Diseases

**Figure 2:**
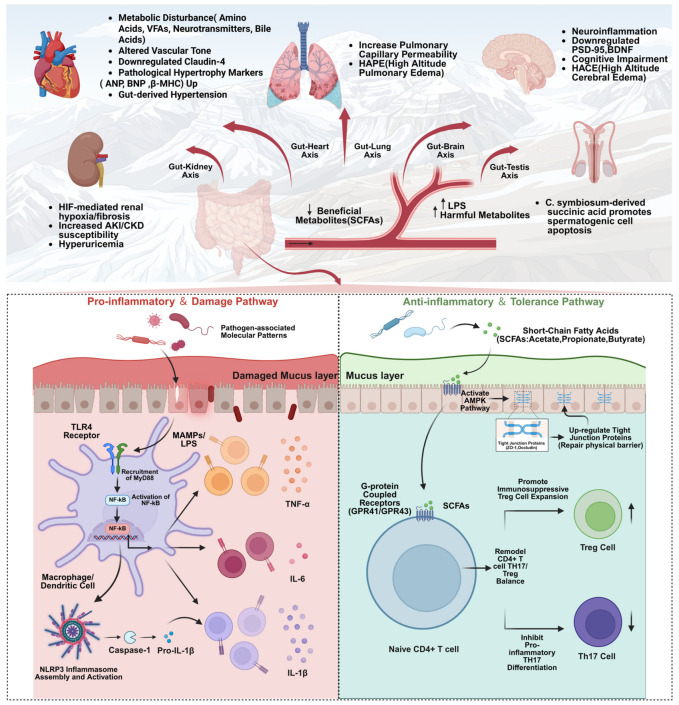
Mechanistic model of gut microbiota-mediated multi-organ crosstalk and immune regulation under high-altitude stress. The (**upper panel**) schematically illustrates how high-altitude environmental hypoxia disrupts the intestinal mucosal barrier, promoting the circulatory translocation of gut-derived lipopolysaccharide (LPS) and pathogenic metabolites, while severely depleting beneficial microbial metabolites, particularly short-chain fatty acids (SCFAs). These systemic pathological signals propagate along distinct physiological axes, leading to multi-organ impairment: the gut–lung and gut–heart axes facilitate increased pulmonary capillary permeability, pulmonary hypertension, and cardiac hypertrophy; the gut–brain axis triggers microglial activation and neuroinflammation, compromising cognitive function; and the gut–kidney and gut–testis axes accelerate renal hypoxia-induced fibrosis and impair male reproductive functions, respectively. The (**lower panel**) summarizes two opposing immune trajectories. The pro-inflammatory/damage pathway is driven by pathogen-/microbe-associated molecular patterns and LPS, activates toll-like receptor 4 (TLR4)/myeloid differentiation primary response 88 (MyD88)/nuclear factor-κB (NF-κB) signaling and the NLR family pyrin domain-containing 3 (NLRP3) inflammasome, and promotes secretion of TNF-α, IL-6, and IL-1β while disrupting the mucus and epithelial barrier. In contrast, the anti-inflammatory/tolerance pathway is mediated by SCFAs, which activate AMP-activated protein kinase (AMPK), upregulate tight-junction proteins (e.g., ZO-1 and occludin), signal through G protein-coupled receptors GPR41/GPR43, and promote regulatory T (Treg) cell expansion while suppressing pro-inflammatory T helper 17 (Th17) differentiation, thereby restoring barrier integrity and immune homeostasis. Created in BioRender. ZP, L. (2026) https://BioRender.com/dmb08vy (accessed on 21 April 2026).

Animal studies further suggest that gut microbiota alterations under high-altitude hypoxia may contribute to host adaptation through microbial metabolites, intestinal barrier regulation, and immune-metabolic responses. Extending beyond localized interactions, high-altitude-associated gut dysbiosis may affect the host through interconnected gut–organ axes. Increased intestinal permeability facilitates the translocation of lipopolysaccharides and other harmful metabolites, whereas reduced levels of beneficial metabolites, particularly short-chain fatty acids, weaken epithelial repair and immune tolerance. The high-altitude stress-driven gut dysbiosis exerts widespread pathological and adaptive effects that extend far beyond localized intestinal interactions. To provide a systemic landscape, Figure 2 conceptualizes this multi-organ crosstalk, stratifying the specific target axes in the upper panel, and delineating the fundamental, bifurcated immune microenvironment (pro-inflammatory signaling vs. SCFA-mediated tolerance pathways) in the lower panel. The detailed molecular cascades and empirical evidence governing each specific organ axis depicted in Figure 2 are thoroughly elaborated in the subsequent subsections (Section 4.1, Section 4.2, Section 4.3, Section 4.4 and Section 4.5).

### 4.1. The Gut–Lung Axis

High-altitude hypoxic diseases such as high-altitude pulmonary edema (HAPE) are highly threatening acute conditions in high-altitude environments, with complex pathogenesis, in which gut microbiota imbalance plays a critical role. First, clinical and animal model studies have both shown that high-altitude hypoxic environments significantly alter the composition and function of the gut microbiota. For example, acute high-altitude hypoxia leads to a reduction in gut microbiota diversity, decreased abundance of probiotics such as *Lactobacillus* and *Muribaculum*, and an increased proportion of potential pathogens [48]. Intestinal microecological dysbiosis in patients with high-altitude pulmonary edema manifests as microbial structural imbalance, accompanied by impaired intestinal mucosal barrier function and increased intestinal permeability; this allows bacteria and their metabolites, such as lipopolysaccharides, to enter the systemic circulation, triggering systemic inflammatory responses and promoting pulmonary edema formation. Recent studies have further revealed that the abnormal enrichment of Klebsiella pneumoniae and Escherichia coli in the gut under hypoxic conditions can induce systemic inflammation and upregulate plasma lysophosphatidylcholines (LPCs) levels, forming a “gut-lung axis” pathogenic pathway. Molecular dynamics simulations and cellular experiments confirm that high levels of LPCs directly disrupt the membrane integrity of human pulmonary microvascular endothelial cells (HPMEC) and human pulmonary alveolar epithelial cells (HPAEpiC), forming physical pores and increasing membrane permeability, thereby exacerbating pulmonary edema [49]. Concurrently, the imbalance of the gut–lung axis is also considered a key driver in the occurrence and development of hypoxic pulmonary hypertension (HPH); the gut microbiota and its mediated metabolic pathways (such as choline metabolism) play a pivotal role in regulating pulmonary vascular tone and remodeling. Recent studies confirm that interventions targeting intestinal microecology can alleviate HPH. For example, the natural flavonoid hesperidin was found to significantly alleviate hypoxia-induced pulmonary hypertension and pulmonary vascular remodeling by restoring gut–lung axis homeostasis and regulating the levels of gut microbiota-derived metabolites [50]. Furthermore, the traditional Tibetan medicine *Oxytropis falcata* Bunge has also demonstrated similar targeted regulatory potential; by reshaping the intestinal microbial community structure and optimizing the metabolite profile, it effectively inhibits pulmonary inflammatory responses and vasoconstriction [51].

### 4.2. The Gut–Heart Axis

Long-term hypoxia exposure can trigger adaptive or pathological changes in the body’s cardiovascular system, among which high-altitude cardiac hypertrophy is a typical manifestation. Pathological myocardial hypertrophy induced by hypobaric hypoxia exposure in rats is accompanied by a significant decrease in the abundance of SCFA-producing gut microbiota (such as *Ruminococcaceae* and *Lachnospiraceae*) and disorders in plasma amino acid and bile acid metabolism. Targeted supplementation with prebiotics or synbiotics can partially reverse intestinal microecological dysbiosis and regulate metabolic abnormalities, thereby significantly alleviating myocardial hypertrophy [52]. Other studies have found that in spontaneously hypertensive rat models, 16S rDNA sequencing revealed a decreased abundance of *Ligilactobacillus murinus* compared with the control group; intragastric administration of this bacterium for two weeks could reverse the decreased expression of claudin-4 in the proximal colon, reduce intestinal permeability, decrease plasma LPS, and ultimately lower systolic blood pressure. These findings provide intervention-based evidence that supplementation with the depleted commensal Lactobacillus may help alleviate hypertensive phenotypes by restoring intestinal barrier integrity and reducing endotoxin translocation, suggesting its potential value as a microbiota-targeted strategy for high-altitude hypoxia-associated enterogenous hypertension [53]. Moreover, gut microbiota metabolites can indirectly regulate the activity of host cytochrome P450 (CYP) family enzymes, altering the metabolism of vasoactive substances such as arachidonic acid, profoundly affecting cardiovascular compensation and pathology [54]. Changes in CYP enzymes not only affect drug metabolism but also alter the levels of endogenous metabolites, subsequently influencing the occurrence and development of cardiovascular diseases. By affecting CYP expression and its metabolic pathways, the gut microbiota may play an important role in the pathogenic mechanisms of high-altitude cardiovascular diseases. Recent research advances indicate that bacteria-derived extracellular vesicles (BEVs) and outer membrane vesicles (OMVs) from the gut microbiota, serving as novel distal communication mediators, play a crucial role in cardiac health. Under high-altitude hypoxic environments, gut microbiota imbalance and the secreted OMVs are considered vital pathways mediating high-altitude myocardial injury. These vesicles, possessing lipid bilayer structures, can traverse the damaged intestinal barrier to enter the systemic circulation and precisely transport encapsulated active substances such as proteins, nucleic acids, and endotoxins to myocardial tissue. Distally in the heart, these vesicles can trigger inflammatory responses, oxidative stress, and cardiomyocyte apoptosis by activating Toll-like receptor (e.g., TLR4, TLR2) signaling pathways, thereby participating in regulating pathological processes of myocardial remodeling, cardiac hypertrophy, and cardiac dysfunction [55,56].

### 4.3. The Gut–Brain Axis

The role of the microbiome-gut–brain axis in high-altitude hypoxic adaptation and related diseases has received widespread attention. Emerging evidence from neurophysiological and gut microbiome studies suggests that gut microbiota remodeling is associated with preserved cerebral function in high-altitude indigenous populations, potentially through gut–brain axis-related mechanisms [57]. Patients with high-altitude cerebral edema also exhibit significant changes in gut microbiota; microbial dysbiosis leads to intestinal barrier disruption, prompting inflammatory factors and bacterial toxins to affect the central nervous system through the gut–brain axis, inducing neuroinflammation and cerebral edema. Studies have found that a reduction in certain key strains, such as *Clostridium* spp., is closely associated with cognitive impairment and brain inflammation; supplementing with specific probiotics can partially restore intestinal barrier function, reduce inflammatory responses, and mitigate brain damage [58]. Extensive animal experiments have shown that chronic high-altitude hypoxia can alter gut microbiota composition, weaken intestinal barrier function, and promote systemic inflammation and oxidative stress, thereby inducing or exacerbating cognitive dysfunction; targeted interventions on the gut microbiota thus hold promise for alleviating high-altitude-related brain injury. Reports indicate that antibiotics exacerbate spatial memory impairment in mice exposed to 4000 m altitude, significantly reducing hippocampal Brain-Derived Neurotrophic Factor (BDNF) and Postsynaptic Density Protein 95 (PSD-95) expression, accompanied by a decrease in ileal *Lactobacillaceae*, an increase in *Corynebacteriaceae*, and a drop in IFN-γ levels [59]. Concurrently, researchers have found that fecal microbiota transplantation (FMT) from high-altitude hypoxic donors to pseudo-germ-free mice can replicate the recipients’ deficits in short-term memory, long-term memory, and contextual fear memory, while simultaneously reproducing elevated intestinal and blood–brain barrier permeability, reduced colonic mucus secretion, and downregulated expression of hippocampal tight junction proteins ZO-1 and Occludin [60]. Other studies point out that in a 5000 m chronic hypoxia model, a pseudo-germ-free state significantly prolongs the escape latency in the Morris water maze, lowers hippocampal BDNF, SYP, and PSD-95 protein levels, and the severity of cognitive impairment is positively correlated with the abundance of *Morganella* and *Klebsiella*, and negatively correlated with *Bifidobacterium* and *Lactobacillus* [61]. Under chronic hypoxic stress, the elevation of the pro-inflammatory factor IL1α activates astrocytes, which in turn inhibits the function of the glutamate transporter SLC1A2, disrupts the balance of excitatory amino acids in the central nervous system, and ultimately leads to pathological damage of DG neurons. This discovery provides an important supplement to gut–brain axis research: systemic inflammatory factors triggered by impaired intestinal barriers and microbial dysbiosis may further activate astrocyte-mediated signaling pathways in the brain via the blood–brain barrier, thereby translating high-altitude environmental stress into central nervous system damage [62]. In summary, targeted reconstruction of intestinal microecology has become a new strategy for alleviating high-altitude cognitive impairment.

### 4.4. The Gut–Kidney Axis

In high-altitude physiological and pathological studies, the gut–kidney axis, as an emerging multi-organ interactive model, provides novel insights for analyzing high-altitude-related kidney injury. Recent studies indicate that systemic stress triggered by high-altitude hypoxic environments significantly compromises intestinal barrier integrity, leading to gut microbiota dysbiosis and metabolic profile alterations. These enterogenous changes exert profound effects on the kidneys via the circulatory system. Specifically, intestinal barrier dysfunction allows massive amounts of gut-derived uremic toxins (such as indoxyl sulfate) and inflammatory mediators to enter the systemic circulation, thereby inducing renal oxidative stress and interstitial fibrosis. In this interactive process, hypoxia-inducible factor (HIF) is confirmed as the core regulatory hub. HIF not only affects barrier function by regulating intestinal epithelial metabolism but also directly participates in the kidneys’ pathological response to hypoxic environments. Under high-altitude exposure, the synergistic effect of abnormal HIF signaling pathway activation and the production of enterogenous toxins co-drives the pathological progression from intestinal dysfunction to kidney damage [63]. Therefore, systemic interventions targeting the gut–kidney axis—including utilizing microecological preparations to repair the intestinal barrier or using pharmacological means to precisely regulate the HIF signaling pathway—have become an important potential therapeutic target for alleviating renal physiological burdens and preventing high-altitude nephropathy in high-altitude environments.

### 4.5. The Gut–Testis Axis

In exploring the impact of high-altitude environments on multi-organ functions, the role of the gut–testis axis in regulating male reproductive homeostasis has gained increasing attention; gut microbiota imbalance caused by high-altitude hypoxia is a significant trigger for reproductive function decline. Long-term exposure to high-altitude environments significantly elevates the abundance of specific gut microbes (such as *C. symbiosum*), leading to a massive accumulation of enterogenous succinic acid and its translocation to testicular tissue via the circulatory system. As a key metabolic signaling molecule, succinic acid can specifically activate the GPR91 receptor on the surface of testicular macrophages, driving macrophage polarization toward a pro-inflammatory phenotype by triggering the GPR91/TRPV4/Ca^2+^ signaling axis. This alteration in the immune microenvironment induces abnormal apoptosis of germ cells and severely impairs sperm quality. This discovery not only establishes succinic acid as another crucial “enterogenous” metabolic messenger—apart from the gut–brain axis—mediating high-altitude injury, but also provides a solid scientific basis for clinical development of strategies to protect the reproductive health of high-altitude exposed populations by regulating gut microbiota homeostasis, inhibiting succinic acid accumulation, or blocking its receptor signaling pathways [64].

Crucially, diet acts as a pivotal primary upstream modulator within these gut–organ axes. While high-altitude hypoxia disrupts the intestinal barrier to promote disease, the intake of specific dietary components can either exacerbate or mitigate this damage. For instance, high-fiber diets provide essential precursors for mitigating systemic inflammation, whereas sudden dietary westernization or malnutrition during altitude travel can compromise mucosal immunity, synergistic with hypoxic stress to accelerate multi-organ pathologies.

## 5. The Association Between Gut Microbiota and Host Immunity

### 5.1. Gut Microbiota-Mediated Immune Adaptation in High-Altitude Environments

The systemic effects of high-altitude-associated gut dysbiosis across multiple gut–organ axes are closely linked to immune–inflammatory regulation [65]. Altered microbial antigens, gut-derived metabolites, and host pattern recognition receptors may jointly shape inflammatory responses, oxidative stress, and immune acclimatization under high-altitude stress. In this context, the gut microbiota can influence intestinal barrier integrity and inflammatory mediator release through metabolites such as SCFAs, thereby contributing to the balance between tissue injury and adaptive compensation [66]. Studies have found that in rats exposed to simulated high-altitude hypoxic environments, intestinal pathogenic bacteria such as *Clostridium* and *Alistipes* are significantly enriched, accompanied by elevated serum pro-inflammatory cytokines such as IL-6 and TNF-α, suggesting that microbial dysbiosis may exacerbate intestinal inflammatory responses by activating mucosal immune responses [67]. Furthermore, acute high-altitude exposure causes a decrease in the abundance of SCFA-producing bacteria such as *Actinobacteriota* and *Enterorhabdus* in mouse intestines, accompanied by elevated serum IL-1β and TNF-α; fecal microbiota transplantation (FMT) can partially reverse these immune abnormalities, providing experimental evidence supporting a causal contribution of microbial dysbiosis [68]. For example, the highly active gut microbial metabolism of the Tibetan chicken at high altitudes enhances the synthesis of SCFAs and secondary bile acids, helping to maintain immune homeostasis and antioxidant capacity, and supporting host energy metabolism and immune stability [69]. In studies on high-altitude pigs, probiotic-fermented feed can significantly increase the abundance of beneficial bacteria like *Lactobacillus*, inhibit opportunistic pathogens, regulate pro- and anti-inflammatory cytokines, and improve immune function [70].

After high-altitude exposure, the abundance of commensal bacteria with immune-protective functions, such as *Akkermansia muciniphila* and *Leuconostoc lactis*, significantly decreases; supplementing with turnip polysaccharides can restore the abundance of these microbes, upregulate L-arginine and L-methionine synthesis pathways, enhance antioxidant enzyme activity, and inhibit the expression of IL-6, IL-1β, and TNF-α, thereby ameliorating hypoxia-induced intestinal barrier damage [71]. Additionally, *Faecalibacterium duncaniae* is significantly enriched during the high-altitude acclimatization phase, and its metabolite 2-ketoglutarate can reduce the apoptosis rate of intestinal epithelial cells by inhibiting the Fos/Nfkbia-mediated apoptotic pathway, maintaining the expression of tight junction proteins ZO-1/Occludin, and subsequently reinforcing the intestinal mechanical barrier and immune homeostasis [72].

### 5.2. Interaction Between Immune Regulation-Related Signaling Pathways and Gut Microbiota

In recent years, research has shown that the gut microbiota engages in complex interactions with the host immune system through various immune regulation-related signaling pathways to control immune homeostasis and inflammatory responses. In high-altitude environments, changes in the structure and metabolites of the gut microbiota subsequently affect the activation of immune signaling pathways, mediating the body’s adaptive responses to high-altitude hypoxia and environmental stress.

Toll-like receptors (TLRs) are vital members of the pattern recognition receptor (PRR) family, capable of recognizing microbe-associated molecular patterns (MAMPs), initiating immune responses, and maintaining intestinal barrier function [73]. The gut microbiota regulates signal transduction in intestinal epithelial cells and immune cells by interacting with TLRs, promoting the maintenance of immune homeostasis [74]. Studies indicate that under the regulation of the gut microbiota, TLRs can promote the intestinal immune system’s tolerance to beneficial bacteria while activating defense responses against pathogens [75,76]. Moreover, the TLR4/MyD88/NF-κB signaling pathway has been proven to be one of the critical pathways through which the gut microbiota regulates immunity. Ginsenoside compounds can modulate the gut microbiota to activate the TLR4/MyD88/NF-κB pathway, enhancing immune function and alleviating immune-related diseases [77]. The NLRP3 inflammasome, serving as another key inflammatory regulator, can sense changes in the gut microbiota and its metabolites, regulate the expression of pro-inflammatory cytokines, and participate in balancing intestinal immune responses [78]. Research has found that milk-derived exosomes can restore intestinal immune homeostasis and alleviate inflammatory bowel disease by inhibiting the TLR4-NF-κB signaling pathway and NLRP3 inflammasome activation [79]. Furthermore, gut microbiota dysbiosis can trigger abnormal TLRs and NLRs signaling pathways, causing intestinal barrier disruption and chronic inflammation, which in turn exacerbates immune response disorders under high-altitude environments [80]. Thus, TLRs and the NLRP3 inflammasome, acting as sentinels of the interaction between the gut microbiota and the host immune system, play a central role in maintaining intestinal immune balance and inflammatory regulation.

### 5.3. The Role of Gut Microbiota Metabolites (SCFAs) in Signal Transduction

It is worth noting that the gut microbiota is shaped not only by physical high-altitude stressors but also significantly by the dietary adaptation required in these environments, such as increased consumption of fiber and protein. Food serves as a primary vehicle through which hosts obtain immunomodulatory components. Dietary fibers and proteins are fermented by altitude-adapted or diet-responsive taxa into key signaling molecules like SCFAs. This dietary–microbial crosstalk modulates the Th17/Treg balance, providing an essential metabolic buffer that restrains hyperactive, hypoxia-induced systemic inflammation and reinforces host immune tolerance. SCFAs not only provide energy for intestinal epithelial cells but also act as signaling molecules to activate G protein-coupled receptors (such as FFAR2/GPR43) and peroxisome proliferator-activated receptor γ, regulating immune cell functions and inflammatory responses [81,82]. Butyrate, in particular, can enhance intestinal barrier function and reduce the release of inflammatory mediators by regulating the expression of tight junction proteins in intestinal epithelial cells [83,84]. Additionally, SCFAs can regulate intestinal immune responses and promote immune tolerance by influencing the Th17/Treg cell balance [85]. Taking the traditional Chinese herbal drink Dendrobium officinale as an example, it improves the metabolic-immune crosstalk of the gut-liver axis by regulating gut microbes and elevating SCFA production to activate the intestinal GPR41/43-ERK1/2 signaling pathway and reshape the Th17/Treg immune homeostasis of CD4+ T cells [86]. Furthermore, gut microbiota metabolites can also regulate signaling pathways such as MAPK and JAK-STAT3, affecting the activation and differentiation of immune cells. For example, certain prebiotic polysaccharides can enhance immune defense by activating the MAPK signaling pathway to increase the expression of anti-inflammatory factors and tight junction proteins [87]. Against the pathological backdrop of high-altitude hypoxia-induced acute injury or chronic inflammation, the integrity of the SCFA-GPR43 axis becomes critically essential to suppress hyperactive Th17-mediated pro-inflammatory responses and restore host immune tolerance [88].

Mechanistically, high-altitude hypoxia-induced gut dysbiosis may impair intestinal barrier integrity and promote LPS translocation. LPS activates the TLR4/MyD88/NF-κB pathway, inducing pro-inflammatory cytokines such as TNF-α, IL-6, and pro-IL-1β. NF-κB also primes NLRP3 inflammasome activation, while hypoxia-related mitochondrial dysfunction, ROS accumulation, and ionic imbalance may further trigger NLRP3 activation, leading to caspase-1-dependent maturation of IL-1β and IL-18 [89,90]. Thus, TLR4/NF-κB and NLRP3 signaling constitute interconnected inflammatory pathways linking microbiota dysbiosis to host inflammatory responses under high-altitude hypoxia.

## 6. The Potential of Microecological Interventions in Promoting High-Altitude Adaptation

Given the potential involvement of gut microbiota and microbial metabolites in high-altitude adaptation, microecological interventions have emerged as a promising strategy to modulate host microecology and prevent altitude-related disorders. Progress in research focusing on microecological intervention methods such as probiotics, prebiotics, and fecal microbiota transplantation has provided new perspectives and technical means for high-altitude adaptation.

Probiotics refer to active microorganisms beneficial to the host, which improve health under high-altitude environments by regulating the gut microbiota, enhancing intestinal barrier function, and boosting immunomodulatory capacity. The supplementation of certain probiotics and prebiotics has been proven to improve gut microbiota structure, enhance intestinal barrier function, and lower inflammatory markers, thereby mitigating the severity of high-altitude hypoxia-related diseases [91,92]. In addition, the regulatory effect of probiotics on the gut microbiome has also been verified in high-altitude poultry farming; although impacts on microbial composition exhibit individual differences, probiotics help maintain intestinal microecological homeostasis and promote animal growth performance and adaptability [93,94].

Prebiotics, serving as a nutritional source for probiotics, can promote the growth and metabolic activity of beneficial intestinal bacteria, indirectly improving the host’s nutritional absorption and immune function. Optimizing dietary strategies to support beneficial microbiota may enhance metabolic, immune, and performance adaptation in high-altitude environments [95,96]. A study found that under hypoxic conditions at 2880 m, inulin as a prebiotic promoted Bifidobacterium and Lactobacillus growth, inhibited *Escherichia coli*, improved intestinal mucosal morphology, and reduced ascites incidence in Haidong chicks, suggesting that it may facilitate adaptation to high-altitude hypoxia by modulating gut microbiota and enhancing intestinal barrier function [97]. Studies on high-altitude animals such as Tibetan pigs, Tibetan antelopes, and Tibetan sheep have found that their gut microbiomes undergo specific metabolic adjustments, such as enhanced SCFA production, due to environmental stress; these metabolites are crucial for energy supply and immune regulation [47,98]. Through reasonable prebiotic supplementation, the activity of these microbial metabolic pathways can be promoted, enhancing the host’s adaptive capacity to high-altitude environments.

Fecal microbiota transplantation (FMT), as a technique to directly modify the host’s gut microbiota composition, has demonstrated significant value in clinical and animal research in recent years. Under high-altitude environments, the diversity and stability of the gut microbiota are challenged, easily leading to health issues such as impaired intestinal barrier function, enhanced inflammatory responses, and acute high-altitude illnesses [99]. FMT helps alleviate high-altitude-related diseases and promotes rapid host adaptation to high-altitude environments by reconstructing a healthy microbiome to restore intestinal barrier integrity and immune homeostasis. Although research on FMT for high-altitude environments is currently in its nascent stage, existing studies have suggested its potential clinical application value, particularly in the prevention and treatment of high-altitude diseases and related immune dysfunctions.

**Figure 3:**
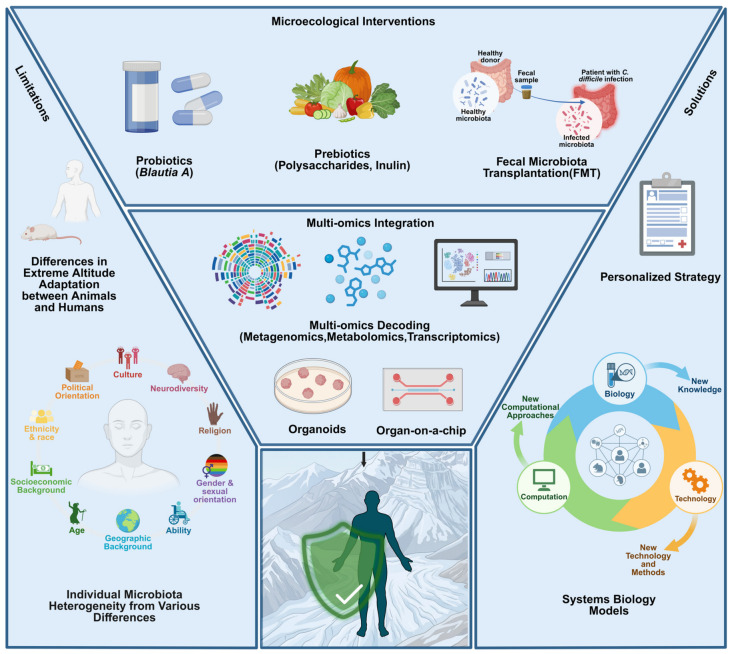
Conceptual framework for microbiota-targeted interventions and translational research to enhance high-altitude adaptation. This translational blueprint outlines strategic modalities targeting the intestinal microecology to mitigate altitude-related physiological disorders and accelerate acclimatization. The targeted populations are clinically stratified into three cohorts requiring tailored regimens: short-term visitors (focusing on acute dysbiosis prevention), long-term immigrants (focusing on gradual metabolic adaptation), and high-altitude residents or indigenous animal models (serving as ecological baselines for adaptive traits). The therapeutic repertoire encompasses: (1) Probiotics (e.g., specific Lactobacillus and Bifidobacterium strains) to reinforce mucosal resistance; (2) Prebiotics and dietary fiber to selectively enrich indigenous SCFA-producing taxa; and (3) Fecal microbiota transplantation (FMT) from adapted high-altitude donors to rapidly re-establish immune–metabolic homeostasis in susceptible hosts. To validate efficacy and safety prior to clinical translation, the framework integrates a comprehensive multi-omics monitoring platform (metagenomics, metabolomics, and host transcriptomics) designed to track critical biomarkers—such as fecal SCFA profiles, circulating LPS levels, and downstream mucosal inflammatory indicators—thereby optimizing population-specific responses and therapeutic timing. Created in BioRender. ZP, L. (2026) https://BioRender.com/dmb08vy (accessed on 21 April 2026).

Microecological regulation strategies have broad application prospects in high-altitude adaptation. First, developing personalized microecological intervention plans tailored to different high-altitude adaptation targets (such as high-altitude residents, high-altitude animals, and high-altitude athletes) will help optimize their intestinal microecological structure and elevate energy metabolism efficiency and immune defense capabilities [100]. Second, utilizing multi-omics technologies (such as metagenomics, metabolomics, and transcriptomics) to deeply analyze the interaction between the gut microbiota and host metabolic, immune, and nervous systems will provide a scientific basis for microecological interventions [101]. From a translational perspective, these findings suggest that the gut microbiota and microbial metabolites may serve as potential biomarkers and intervention targets for high-altitude adaptation and high-altitude-related diseases. For short-term visitors to high-altitude environments, microbiota-targeted interventions may help alleviate gut microbiota dysbiosis, intestinal barrier disruption, and inflammation under high-altitude hypoxia. For long-term immigrants and high-altitude residents, personalized probiotics, prebiotics, or FMT may support stable gut microbiota remodeling and immune–metabolic homeostasis. In addition, microbial metabolites and host-response indicators, including SCFAs, LPS, bile acids, succinic acid, intestinal barrier integrity, and inflammatory pathways such as TLR4/MyD88/NF-κB and NLRP3, may be useful for evaluating high-altitude adaptation and intervention efficacy. However, before clinical application, future studies should identify core microbial effectors, validate metabolite-based biomarkers, and conduct well-designed randomized controlled trials to assess efficacy, safety, optimal dosage, and population-specific responses. Lastly, future research can further explore the optimal methods for probiotic strains, prebiotic components, FMT, and their combined application effects, offering more effective microecological regulatory strategies for high-altitude adaptation (Figure 3).

## 7. Conclusions

High-altitude hypoxia is closely associated with changes in the gut microbiota, microbial metabolites, intestinal barrier function, and host immune–metabolic responses. Current evidence from human and animal studies suggests that the gut microbiota may participate in high-altitude adaptation and the development of high-altitude-related diseases by regulating energy metabolism, inflammation, oxidative stress, and barrier integrity. In particular, microbial metabolites such as short-chain fatty acids, bile acids, succinic acid, and lipopolysaccharide may serve as important mediators linking environmental hypoxia with host physiological responses.

Although growing evidence supports the involvement of the gut microbiota in high-altitude adaptation, the field remains at an early stage. Most existing studies are descriptive or based on limited cohorts, and causal relationships between specific microbial taxa, metabolites, and host outcomes remain insufficiently defined. Future research should integrate multi-omics approaches, longitudinal cohort studies, germ-free or fecal microbiota transplantation models, and well-designed clinical trials to identify core microbial effectors and validate reliable biomarkers.

Overall, the gut microbiota represents a promising but still underexplored target for understanding and improving adaptation to high-altitude environments. Microbiota-targeted strategies, including probiotics, prebiotics, dietary modulation, and fecal microbiota transplantation, may offer potential approaches for preventing or alleviating high-altitude-related disorders. However, their efficacy, safety, optimal timing, and population-specific effects require further rigorous investigation before clinical application.

## Figures and Tables

**Table 1:** Human studies on gut microbiota responses to high-altitude exposure or adaptation.

Study Population/Condition	Major Microbiota Changes	Functional/Metabolic Changes	Key Conclusion	Reference
Tibetans from the Qinghai–Tibet Plateau vs. Han Chinese; cross-sectional; 16S rRNA sequencing plus metabolomics.	Han Chinese were dominated by *Bacteroides* and *Bifidobacterium*, whereas Tibetans were dominated by *Bacteroides* and *Prevotella*; *Pseudomonas*, *Prevotella*, and other taxa differed significantly.	Differential metabolites were mainly ferulic acid and 4-methylcatechol; alanine aminotransferase and uric acid were strongly correlated with differential taxa and metabolites.	Stable differences in gut microbial composition and metabolic features exist between Tibetans and Han Chinese living under long-term plateau conditions, and *Prevotella* may be related to Tibetan high-altitude adaptation.	[17]
Native Tibetan herders at 3900 m vs. Han migrants living at the same altitude; cross-sectional; 16S rRNA sequencing plus hematology.	Tibetans showed higher alpha diversity and a community structure distinct from that of Han migrants.	Tibetans had higher platelet counts; Megamonas was positively associated with body mass index, *Bifidobacterium* was negatively associated with body mass index, and *Alistipes*/*Parabacteroides* were positively associated with platelet count; predicted functions were more oriented toward carbohydrate metabolism.	The microbiota structure of native Tibetans is associated with hematologic adaptive phenotypes, suggesting that gut microbiota may participate in high-altitude adaptation and acclimatization.	[19]
High-altitude Tibetan patients with coronary artery disease, healthy Tibetans, and high-altitude or lowland Han patients with coronary artery disease and healthy controls; 16S rRNA sequencing.	High-altitude Tibetan patients with coronary artery disease showed higher alpha diversity; *Catenibacterium*, *Clostridium_sensu_stricto*, *Holdemanella*, and *Ruminococcus 2* were enriched; *Prevotella* was more abundant than in Han patients with coronary artery disease.	*Catenibacterium* showed a positive correlation with *Prevotella*. In addition, *Catenibacterium*, *Holdemanella*, and *Prevotella* were positively associated with the intake of fermented dairy products, carbohydrates, and dietary fiber.	Even under a disease background, high-altitude Tibetans retained microbial patterns linked to altitude and diet, and these microbial changes were closely related to dietary structure.	[20]
Lowland Han Chinese, Han Chinese after 4 days, 6 days, or 3 months at high altitude, Han Chinese 3 months after returning to low altitude, and long-term resident Tibetans; 393 healthy adult men; 16S rRNA sequencing plus 76 blood indices.	The high-altitude environment reshaped the Han gut microbiota within 4 days and for more than 3 months; with longer residence, Han microbial profiles gradually approached those of Tibetans; after 3 months back at low altitude, the profiles still resembled the high-altitude group more than the lowland baseline.	The high-altitude group had lower blood glucose and higher testosterone; glucose showed Tibetan-specific associations with *Succinivibrio* and *Sarcina*.	High-altitude hypoxia exerts rapid and persistent effects on gut microbiota and clinical indices, and this remodeling does not quickly or completely reverse after leaving high altitude.	[25]
The same cohort of 45 Han adults moving from 243 m to 3658 m and then back; 108-day longitudinal metagenomics; validated in another Han cohort with long-term exposure at 4700 m.	After high-altitude exposure, microbial species and functional diversity decreased whereas functional redundancy increased; the changes were driven mainly by overgrowth of *Blautia A*.	*Blautia A* was also enriched in the long-term high-altitude Han cohort; animal experiments suggested anti-inflammatory and gut-barrier protective roles.	A reproducible core responder taxon, *Blautia A*, appears during high-altitude acclimatization and may promote adaptation to hypoxia by maintaining intestinal health.	[24]
Han Chinese who moved from lowland to Lhasa and were followed up at 3, 6, and 12 months, with Tibetan controls included; 16S rRNA sequencing.	High altitude altered gut microbial diversity and composition; with longer residence, the microbiota composition and abundance of migrant Han participants approached a new steady state.	Symptoms of irritable bowel syndrome were significantly alleviated during follow-up; IBS-related *Alistipes*, *Oscillospira*, and *Ruminococcus torques* were more abundant in the high-altitude IBS subgroup.	High-altitude-related dysbiosis is associated with irritable bowel syndrome and psychosocial abnormalities, but some symptoms may improve after long-term exposure.	[29]
406 healthy adult men; baseline at 800 m, then 7 days at 4500 m, followed by 2 weeks at low altitude after 3 months at high altitude; longitudinal 16S rRNA sequencing.	Acute high-altitude exposure exerted stronger effects on alpha and beta diversity; opportunistic taxa such as *Ruminococcus* and *Oscillibacter* increased, whereas beneficial short-chain-fatty-acid-producing taxa such as *Faecalibacterium* and *Bifidobacterium* decreased.	These changes persisted after return to low altitude, suggesting long-lasting remodeling; functional pathways indicated metabolic adjustments related to energy utilization.	Acute plateau hypoxia first induces marked dysbiosis, whereas sustained exposure further drives structural and functional remodeling with a lagged effect.	[28]
Volunteers from Guangzhou to Tibet exposed from below 50 m to 2900 m for 12 months; metagenomics plus serum metabolomics.	Enterotypes could shift from a *Blautia*-dominated type to a *Bacteroides*-dominated type; the proportion of *Bacteroides*-dominated individuals increased after exposure.	The *Bacteroides*-dominated type showed lower TNF-alpha; participants converting from the *Blautia*-dominated type to the *Bacteroides*-dominated type showed improved fasting glucose, increased *Bacteroides thetaiotaomicron* and *Bacteroides uniformis*, decreased *Escherichia coli*, and reduced L-glutamate.	Moderate-altitude exposure can induce enterotype conversion, and a *Bacteroides*-dominated enterotype may correspond to more favorable metabolic and inflammatory phenotypes.	[27]
70 healthy young and middle-aged individuals; followed for 6 months after moving from about 1600 m to 4500 m; 16S rRNA sequencing plus fecal metabolomics.	The high-altitude group showed lower alpha diversity and overall abundance than the low-altitude group; differences in *Firmicutes* and *Blautia* were most pronounced; *Actinobacteriota*, *Blautia*, *Bifidobacterium*, *Fusicatenibacter*, and *Anaerostipes* showed higher relative abundance.	Large numbers of upregulated and downregulated metabolites were detected, involving six classes of KEGG pathways.	After 6 months at high altitude, gut microbiota diversity, composition, and metabolomic profile changed significantly, supporting coordinated microbiota-metabolite adaptation to long-term hypoxic exposure.	[26]

**Table 2:** Animal and cross-species studies on gut microbiota responses to high-altitude exposure or adaptation.

Study Population/Condition	Major Microbiota Changes	Functional/Metabolic Changes	Key Conclusion	Reference
Fourteen days in a hypobaric hypoxia chamber; comparison among control, high-altitude exposure, and high-altitude exposure plus probiotic groups.	High-altitude exposure induced gut dysbiosis; after supplementation with *L. johnsonii* YH1136, *Lactobacillus* became dominant and pathogenic bacteria decreased; *Staphylococcus* and *Corynebacterium 1* were associated with changes in ileal microRNAs.	*L. johnsonii* YH1136 ameliorated gut-barrier injury and reduced the risk of pathogen translocation across the barrier.	Acute high-altitude hypoxia can induce gut dysbiosis and intestinal injury in mice, whereas *L. johnsonii* YH1136 derived from high-altitude humans can alleviate these changes by reconstructing the microbiota.	[31]
Fecal comparison among three Tibetan regions with different altitudes and temperatures (DXS, LZS, and NMS).	The bacterial community was dominated by *Firmicutes*, *Bacteroidota*, and *Actinobacteriota*; dominant genera included *UCG-005*, Christensenellaceae_R-7_group, and Rikenellaceae_RC9_gut_group; 32 bacterial genera and four fungal genera differed significantly among regions.	Predicted digestive-system-related functions were more abundant in the NMS group, along with differences in lipid transport and metabolism and inorganic ion transport.	Yak gut microbiota undergoes structural and functional remodeling under different plateau altitude and temperature conditions, suggesting that environmental gradients are major drivers of adaptation.	[34]
High-altitude yak model simulating warm-season grazing, winter nutritional stress, and winter supplementary feeding.	All three groups were dominated by *Bacteroidetes* and *Firmicutes*; *Actinobacteriota*, *Succinivibrionaceae*_UCG-002, and *Ruminococcus albus* were more abundant in warm-season grazing; *Bacteroides* was higher under winter nutritional stress; *Neocallimastix* was higher under winter nutritional stress and winter supplementary feeding, and *Cyllamyces* was more abundant under winter nutritional stress.	Acetate, propionate, and total volatile fatty acids were significantly higher during warm-season grazing, indicating synchronized enhancement of nutrient supply and rumen fermentation intensity.	Plateau ruminants can adapt to seasonal fluctuations in forage quality by remodeling bacterial, fungal, and archaeal communities.	[35]
Comparison between high-altitude Tibetan cashmere goats (4750 m) and low-altitude Jianzhou big-eared goats (500 m).	The high-altitude group showed coupled changes between gut microbiota and liver metabolites; *Andreesenia*, *Dielma*, *Oscillibacter*, *Agrobacterium*, *Hyella* and *Thermosinus* were reported to be associated with high-altitude adaptation.	Liver transcriptomics and metabolomics indicated altered glycerophospholipid, retinol, and related pathways, together with enhanced immune regulation and energy-use efficiency.	From a liver–gut axis perspective, gut microbiota changes in high-altitude goats are not isolated phenomena but act jointly with host metabolism and gene expression in plateau adaptation.	[37]
Comparison of cecal contents across different altitude gradients on the Qinghai–Tibet Plateau.	With increasing altitude, microbiota diversity and co-occurrence network complexity decreased; *Bacteroidetes* increased whereas *Firmicutes* decreased.	Overall short-chain fatty acid levels remained stable, but KEGG pathways related to metabolism, genetic information processing, and organismal systems were upregulated, and community assembly became more deterministic.	Higher altitude does not necessarily increase microbial diversity; in plateau pikas, a more prominent feature is community simplification with functionally directed enhancement.	[39]
Comparison of gut microbiota between summer and extremely cold winter.	Gut microbial diversity decreased in winter, and some fiber-degrading taxa such as *Oscillospira* and *Treponema* declined.	Cellulase activity and total short-chain fatty acids decreased in winter, and predicted abundances of metabolism-related functions, such as energy and lipid metabolism, were lower.	Wild plateau pikas adapt to resource-limited severe winter not by simply enhancing fermentation, but by reducing part of their fermentative and energetic demands.	[38]
Comparison of gut microbiota across high and low altitude and winter and summer, combined with fecal microbiota transplantation.	Altitude significantly affected *Firmicutes* and *Proteobacteria*; *Lactobacillus* was markedly increased in high-altitude winter tree sparrows, and *Clostridium sensu stricto* 1 was also a dominant taxon under high-altitude winter conditions.	Fecal microbiota transplantation showed that a high-altitude microbiota can enhance avUCP (avian Uncoupling Protein) and upregulate thermogenesis-related genes such as syt1 and chodl.	Tree sparrows may use seasonally enriched *Lactobacillus* and related taxa to promote thermogenesis and energy conservation, thereby adapting to high-altitude environments.	[40]
Integrated 16S and 16S rRNA, metagenomic, and transcriptomic analyses of pika gut communities across altitudes.	Active taxa were represented by *Oscillospira* and *Ruminococcus*; *Firmicutes* carried most cellulase and hemicellulase genes, and *Treponema* also participated in polysaccharide degradation.	Cellulose and hemicellulose metabolic pathways were upregulated in the middle- and high-altitude groups; lignin-metabolism genes showed the highest expression proportion, followed by cellulose- and hemicellulose-metabolism genes.	Plateau pikas improve energy acquisition from herbivory by strengthening plant-polysaccharide degradation systems, representing a typical example of functional microbial adaptation in high-altitude herbivores.	[41]
Comparison of gut microbiota among different host species under the same high- and low-altitude environments.	Alpha diversity was generally higher in high-altitude humans, wild macaques, and dogs than in their low-altitude counterparts; under high-altitude conditions, UniFrac distances among the three hosts were smaller, and the number of shared core amplicon sequence variants increased to 5.34 times that at low altitude.	Microbial convergence was more pronounced at high altitude, and neutral models suggested stronger effects of cross-host dispersal and stochastic processes.	High-altitude pressure alters not only single-host microbiota but also promotes convergent evolution of gut microbiota across species; dietary similarity may further strengthen this convergence.	[43]
Multi-site gastrointestinal metagenomic comparison between high-altitude Tibetan pigs and low-altitude black pigs.	Lactobacillus, Bifidobacterium, *Megasphaera*, Fusobacterium, and *Mitsuokella* were more abundant in Tibetan pigs, whose microbial network was also more complex and stable.	Propionate and butyrate metabolism pathways were enriched, together with stronger enzymatic signatures related to fatty acid metabolism and the tricarboxylic acid cycle.	The gastrointestinal microbiota of Tibetan pigs may support energy supply under hypoxic conditions by enhancing short-chain fatty acid production and carbohydrate metabolism.	[44]
Multi-omics comparison among wild winter, wild summer, and captive conditions.	Winter enriched short-chain-fatty-acid-associated taxa such as *Rikenellaceae*_RC9_gut_group and unclassified_c_*Clostridia*, whereas captivity enriched potential pathogens and taxa with fewer short-chain-fatty-acid-producing members, such as *Cellulosilyticum* and *Paeniclostridium*.	Butyrate, valerate, and isovalerate were higher in winter; carbohydrate metabolism, amino acid metabolism, and immunoregulatory pathways were enhanced in winter.	In high-altitude white-lipped deer, extreme winter exerts stronger effects on microbiota composition and metabolism than captivity, and short-chain-fatty-acid-associated taxa are closely linked to winter adaptation.	[45]
Integrated analysis of rumen 16S rRNA sequencing, volatile fatty acids, epithelial morphology, and transcriptomics in Tibetan sheep across low, middle, and high altitudes.	Rumen microbial diversity decreased with altitude; *Rikenellaceae*_RC9_gut_group and *Ruminococcaceae*_NK4A214_group increased with altitude.	The acetate-to-propionate ratio decreased; lipid metabolism functions were enriched in the high-altitude group, accompanied by higher IgA and IgG and increased rumen papilla width and basal-layer thickness.	Tibetan sheep coordinate fermentation metabolism and epithelial immune-barrier function through rumen microbiota-host gene interactions under high-altitude conditions.	[47]

## Data Availability

No new data were created or analyzed in this study.
